# Circulating Serum Micro-RNA as Non-Invasive Diagnostic Biomarkers of Endometriosis

**DOI:** 10.3390/biomedicines12102393

**Published:** 2024-10-19

**Authors:** Antonella Ravaggi, Cosetta Bergamaschi, Chiara Galbiati, Laura Zanotti, Aline S. C. Fabricio, Massimo Gion, Elia Cappelletto, Antonette E. Leon, Massimo Gennarelli, Cesare Romagnolo, Giuseppe Ciravolo, Stefano Calza, Eliana Bignotti, Franco Odicino

**Affiliations:** 1 Department of Obstetrics and Gynecology, ASST Spedali Civili di Brescia, University of Brescia, 25123 Brescia, Italy; c.bergamaschi001@studenti.unibs.it (C.B.); laura.zanotti@asst-spedalicivili.it (L.Z.); ciravolog@gmail.com (G.C.); bignottieliana@gmail.com (E.B.); franco.odicino@unibs.it (F.O.); 2 Department of Clinical and Experimental Sciences, University of Brescia, 25123 Brescia, Italy; 3 Angelo Nocivelli Institute of Molecular Medicine, ASST Spedali Civili di Brescia, University of Brescia, 25123 Brescia, Italy; 4 Residency Program for Clinical Pathology and Clinical Biochemistry, University of Brescia, 25123 Brescia, Italy; 5 Department of Theoretical and Applied Sciences, eCampus University, 22060 Novedrate, Como, Italy; c.galbiati@fatebenefratelli.eu; 6 Basic and Translational Oncology, Veneto Institute of Oncology IOV-IRCCS, 35128 Padua, Italy; alinesueli.coelhofabricio@iov.veneto.it (A.S.C.F.); elia.cappelletto@iov.veneto.it (E.C.); 7 Regional Center for Biomarkers, Department of Clinical Pathology, AULSS3 Serenissima, 30122 Venice, Italy; massimo.gion@aulss3.veneto.it (M.G.); antonette.leon@aulss3.veneto.it (A.E.L.); 8 Division of Biotechnology, Department of Molecular and Translational Medicine (DMTM), University of Brescia, 25123 Brescia, Italy; massimo.gennarelli@unibs.it; 9 Unit of Obstetrics and Gynecology, Dell’Angelo Hospital, Via Paccagnella 11, 30174 Mestre, Italy; cesare.romagnolo@gmail.com; 10 Unit of Biostatistics and Bioinformatics, Department of Molecular and Translational Medicine, University of Brescia, 25123 Brescia, Italy

**Keywords:** endometriosis, microRNA, expression profile, liquid biopsy

## Abstract

Background/Objectives: Endometriosis (END) is a painful gynecological condition. Clinical examination, imaging, and laparoscopy can provide a definitive diagnosis of END. Nonetheless, non-invasive biomarkers could help enhance and streamline the diagnostic process. Micro-RNAs (miRNAs), a family of small non-coding RNAs, could serve as useful non-invasive biomarkers for END. The aim of this study was to perform serum miRNA profiling in a retrospective cohort of women to identify miRNAs that are differentially expressed in END compared to control patients. Methods: RNA was isolated from serum samples of 67 END patients and 60 control women. The expression profile of a 754-miRNA panel was studied with RT-qPCR performed on a QuantStudio 12K Flex with the TaqMan OpenArray miRNA panel. A Censored Regression Model was used for miRNA differential expression analysis. Several gene-enrichment algorithms were employed to identify pathways related to the target genes of differentially expressed miRNAs. Results: One hundred and thirty miRNAs were detected in at least 75% of samples from either the END or the control group. Sixteen miRNAs were significantly modulated between the END and control groups. Enrichment analysis identified targets significantly overrepresented in numerous pathways involved in biological processes related to END, including inflammation, angiogenesis, cellular invasion, cell-cycle/cell proliferation, and estrogen and progesterone hormonal signaling. Conclusions: Our study indicates that differentially expressed miRNAs between END patients and controls can be identified through liquid biopsy. Our findings also suggest a potential role for serum miRNAs in the pathophysiology of END, warranting further investigations for their use as non-invasive biomarkers.

## 1. Introduction

Endometriosis (END) is a debilitating gynecological disorder, characterized by the presence of endometrial-like cells in ectopic sites outside the uterine cavity throughout the pelvis, primarily in the ovaries (forming endometriomas), peritoneum, rectum, rectosigmoid, rectovaginal pouch, and uterosacral ligaments. In rare cases, END lesions can also affect organs outside the genital tract, such as the pericardium, pleura, and diaphragm [1,2,3], and can manifest either as a superficial or deep infiltrating endometriosis (DIE). END is a high-prevalence disease, affecting over 176 million women worldwide [4]. It has an incidence range of 6–10% among the general female population during reproductive age, which rises to 35–50% in women with infertility [3]. Clinical END-associated symptoms impact multiple body sites and heavily affect women’s quality of life, particularly work and sexual sphere [2,4,5,6]. Moreover, several reports suggest that women with END may have an increased risk of developing malignant pelvic tumors, mainly endometrioid or clear-cell ovarian carcinoma, a lethal form of gynecological cancer [3]. END diagnosis can be challenging due to the broad spectrum of symptoms, which may vary in severity or be entirely absent, making misdiagnosis common. These diagnostic issues can lead to a 4 to 12-year delay from the onset of symptoms to diagnosis, significantly affecting patients’ psychological well-being [7]. Transvaginal ultrasound is the imaging technique used to detect END, while magnetic resonance imaging serves as a complementary tool option [8,9]. However, a definitive diagnosis of END can only be made through laparoscopy, confirmed by histological evaluation of tissue biopsy [7,10]. Despite its diagnostic accuracy, laparoscopy is invasive, and it is often postponed or avoided in young women, leading to a delay in diagnosis and treatment, which can exacerbate symptoms over time [4,11]. Thus, the discovery of non-invasive biomarkers is crucial to address the limitations of current diagnostic approaches.

Liquid biopsy, a minimally invasive procedure involving the analysis of biomarkers in body fluids, offers a cost-effective and repeatable alternative to traditional tissue biopsies, enabling better monitoring of disease evolution and treatment response [12]. Several types of circulating biomarkers have been investigated as potential diagnostic indicators of END, including glycoproteins, inflammatory molecules, oxidative stress markers, autoantibodies, growth factors, molecules involved in angiogenesis, urinary peptides, and micro-RNAs (miRNAs). However, no single molecule or combination has yet achieved sufficient sensitivity and specificity to be validated for clinical use in END patients [1,13]. Among the various biomolecules, miRNAs have emerged as particularly promising non-invasive biomarkers for END diagnosis [1,14]. Approximately 2000 miRNAs have been identified in humans, regulating about one-third of all genes [15] and controlling a wide range of biological processes, including implantation, embryo development, angiogenesis, inflammation, cell differentiation, cell cycle, and tissue remodeling. These are functions related to endometrial functions and the development of gynecological diseases, including END [12,16]. MiRNAs are present in all tissues, but they can also be released into circulation, where they may play a role in communication between the eutopic endometrium and ectopic endometrial lesions, causing dysregulation of gene expression [14,17].

Given their potential involvement in the pathophysiology of END and their high stability in both body fluids and tissue, miRNAs have been the subject of several studies aimed at determining their utility as biological markers for diagnosing END [10,18,19,20,21,22,23,24,25,26]. However, while initial findings are encouraging, the results are still controversial, and no specific miRNAs or panel of miRNAs have yet been validated for clinical use.

The aim of this study was to perform serum miRNA profiling using the TaqMan OpenArray^®^ system on a cohort of patients with and without END. Our study identified a panel of miRNAs differentially expressed between END and control groups, as well as between DIE and endometriomas (OMA), suggesting that serum miRNAs may be used as diagnostic biomarkers of END and play a role in the pathophysiology of this disease.

## 2. Materials and Methods

### 2.1. Patients and Biological Samples

This retrospective multicenter study was performed on serum samples of 67 patients with END and 60 controls (CNT), diagnosed and treated at the Departments of Obstetrics and Gynecology of “ASST Spedali Civili di Brescia” and “Azienda ULSS3 Serenissima”, from January 2009 to February 2012. The study was conducted in accordance with the Declaration of Helsinki set of principles and was approved by both the Research Review Boards and Ethics Committees of Brescia (study reference number: NP5015) and “Provincia di Venezia e IRCCS San Camillo” (study reference Prot: FREEDOM TRIAL). All patients had undergone surgery and histological diagnosis and were divided into patients with END and CNT with benign gynecological pathologies not associated with END. Clinicopathologic characteristics of patients with END and CNT are described in Table 1.

A fasting blood sample was collected before surgery and on the same day in a SST Vacutainer Becton Dickinson tube (Franklin Lakes, NJ, USA). The tube was allowed to stand at room temperature for 2 h ± 15 min and centrifuged at 1500× *g* for 10 min to separate the serum, which was then aliquoted and immediately stored at −80 °C in the biorepositories of Brescia and Mestre-Venice Hospitals. Aliquots were kept frozen and never thawed before this study.

### 2.2. RNA Extraction

To extract RNA, the samples were thawed at room temperature. Total RNA was extracted from 400 μL of serum with the miRNeasy Serum/Plasma Advanced Kit (Qiagen, Hilden, Germany), according to the manufacturer’s instructions. RNA was eluted in 22 μL of nuclease-free water and quantified using the Qubit micro-RNA assay kit (Thermo Fisher Scientific, Waltham, MA, USA) by the Qubit 4 Fluorometer (Thermo Fisher Scientific).

### 2.3. Serum miRNA Profiling by TaqMan OpenArray^®^ System

The cDNA templates were prepared using the TaqMan Advanced miRNA cDNA Synthesis Kit (Thermo Fisher Scientific) according to the manufacturer’s guidelines, starting from 10 ng of RNA. Briefly, the workflow of the procedure includes: (i) addition of a 3′ poly(A) tail, (ii) 5′ adapter ligation, (iii) reverse transcription reaction with a universal primer. Following the protocol recommendations, we performed a 22-cycle pre-amplification step with 5 μL of cDNA in a final volume of 50 μL. The miR-Amp reaction products were stored at −20 °C until they were used for the RT-qPCR (within one month).

The miR-Amp reaction products were thawed on ice and then diluted 1:20 in 0.1x TE buffer pH 8 (10 mM Tris, 1 mM EDTA, Thermo Fisher Scientific). Each RT-qPCR mix was prepared by combining equal quantities of a 1:20 diluted sample and 2x TaqMan OpenArray Real-Time PCR Master Mix (Thermo Fisher Scientific) in a final volume of 60 μL. Each sample mix was transferred in 16 wells (6 μL/well) of an OpenArray 384-well Sample Plate (Thermo Fisher Scientific) and then automatically loaded into a Fixed-content TaqMan OpenArray Plate containing the Human Advanced miRNA panel (Cat. N. A32710, Thermo Fisher Scientific) by the QuantStudio™ 12K Flex Accufill System (Thermo Fisher Scientific). Each OpenArray plate was loaded with three samples and run on the QuantStudio 12K Flex Real-Time PCR System v1.3 (Thermo Fisher Scientific). The quantification cycle (Cq) was automatically calculated using the ThermoFisher Cloud Software: Thermo Fisher Scientific Relative Quantification Analysis Module v1.1, available online at https://www.thermofisher.com/de/de/home/cloud.html, accessed on 19 April 2023. Only target values meeting all the following criteria were considered for subsequent analyses: Amplification Score > 1, Cq confidence > 0.8, Cq ≥ 6. MiR-16-5p, was spotted 16 times in each sub-array and was used as a quality control of the OpenArray procedure.

Each serum sample was evaluated for the presence of hemolysis. The ratio between the expression levels of miR-451a (expressed in red blood cells) and miR-23a-3p (not affected by hemolysis) was calculated and samples with a delta Cq < 8 were considered acceptable [27]. Samples with hemolysis were excluded from the study.

### 2.4. Data Analysis

#### 2.4.1. miRNA Preprocessing

In order to allow unbiased comparisons among groups, we opted for applying a reference-based normalization. Therefore, the first step was the identification of the best reference miRNAs among the whole set. Different methods (BestKeeper, NormFinder, Genorm, and the comparative delta-Ct method) were used for estimating optimal reference miRNAs using the RefFinder algorithm [28]. Each algorithm assigns a rank to the miRNAs and an overall rank is determined. An additional algorithm, the Two-One-Sided Test (TOST) [29] was considered to test the equivalence of each miRNA between the END and control groups.

In order to implement the different algorithms, it was necessary to impute the missing data. The imputation procedure was conducted as follows: (a) a right-truncated distribution was considered for each miRNA (i.e., the presence of a detection upper limit), (b) the maximum observed value, on which the upper truncation value was defined, was equal to Ct = 40, (c) to avoid introducing spurious homogeneity between the groups, the mean and standard deviation (SD) of each miRNA within the group (END and CNT) were estimated using a censored regression model (taking into account the truncation of the data) [30], (d) for each missing datum in each group, a random value extracted from a truncated normal distribution was generated, with the mean and SD estimated in the previous step. Among the miRNAs with greater stability based on RefFinder and TOST, miRNAs amplified in at least 95% of the samples were selected. The arithmetic mean of the three most stable miRNAs (reference miRNAs) was used for the subsequent normalization. As a final step, the imputed values were set to missing again before downstream analysis.

#### 2.4.2. miRNA Differential Expression Analysis

In order to evaluate the miRNA expression levels in serum samples, miRNA expression was determined using the 2^−ΔΔCt^ (RQ) relative quantification method: Fold Change (FC) = log_2_RQ_END_/log_2_RQ_CNT_ [31]. Only miRNA with at least 75% amplification in either group were considered. The Censored Regression Model [30] was used for miRNA differential expression analysis, assuming that missing values are censored (they have a value, but this is unknown).

#### 2.4.3. Pathway Analysis

MiRNAs were first matched to annotated gene targets based on multiple databases using the multiMIR R package [32]. Only validated miRNA-gene annotations were considered. Several gene-enrichment algorithms were adopted: Gene Ontology (GO) Biological Processes (GO-BP) and Molecular Function (GO-MF) annotations, Kyoto Encyclopedia of Genes and Genomes (KEGG), Reactome, and WikiPathways [33]. An additional procedure was adopted using the miRNET online application [34].

All analyses were performed using R [https://www.R-project.org/] version 4.4.0.

## 3. Results

### 3.1. Serum miRNA Profiles

We conducted an OpenArray based serum profiling, which allows the detection of 754 human miRNA targets, on 67 END and 60 CNT. One hundred and thirty out of seven hundred and fifty-four miRNAs were detected in at least 75% of samples of either group and were considered for subsequent analyses. Data were then normalized using the arithmetic mean of the three best reference miRNAs: miR-15a-5p, miR-26b-5p, and miR-92a-3p.

We performed four comparisons: (1) all END versus CNT, (2) DIE versus CNT, (3) OMA versus CNT, and (4) DIE versus OMA. Lists of differentially expressed miRNAs between groups are shown in Table 2 and Table 3.

### 3.2. Identification and Functional Analysis of miRNA Target Genes

**Figure 1:**
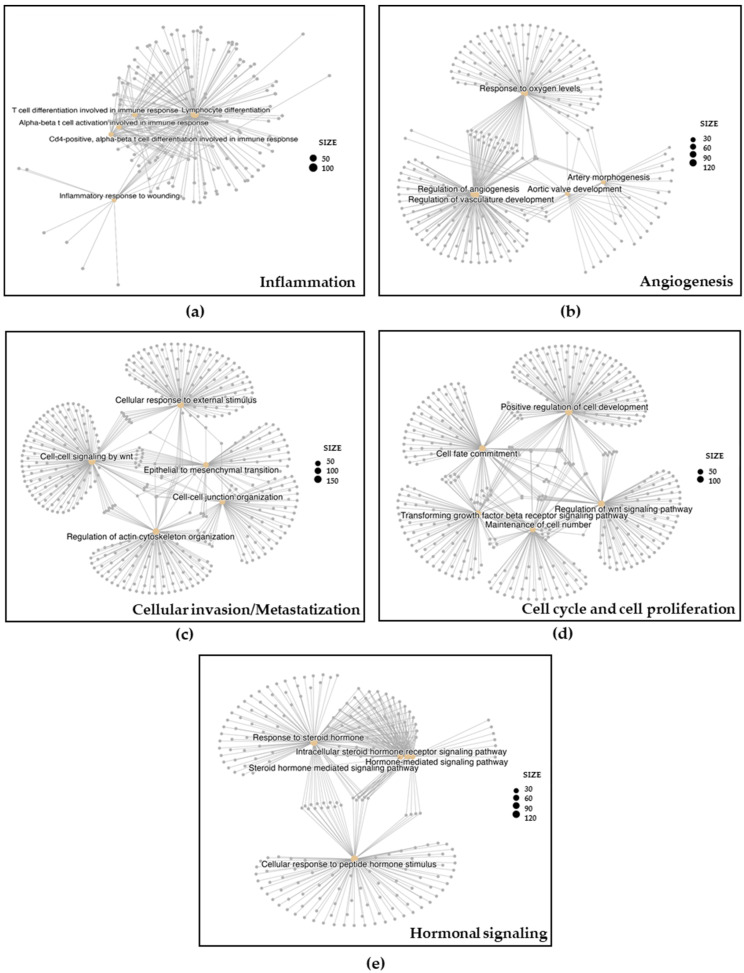
Gene-concept networks for selected Gene Ontology Biological Processes (GO-BP) linked to differentially expressed miRNA between endometriosis patients and controls. Pathways were grouped based on their involvement in the main biological processes associated with endometriosis: inflammation (**a**), angiogenesis (**b**), cellular invasion/metastatization (**c**), cell cycle and cell proliferation (**d**), and hormonal signaling (**e**). Dot size represents the number of genes involved in the different pathways.

**Figure 2:**
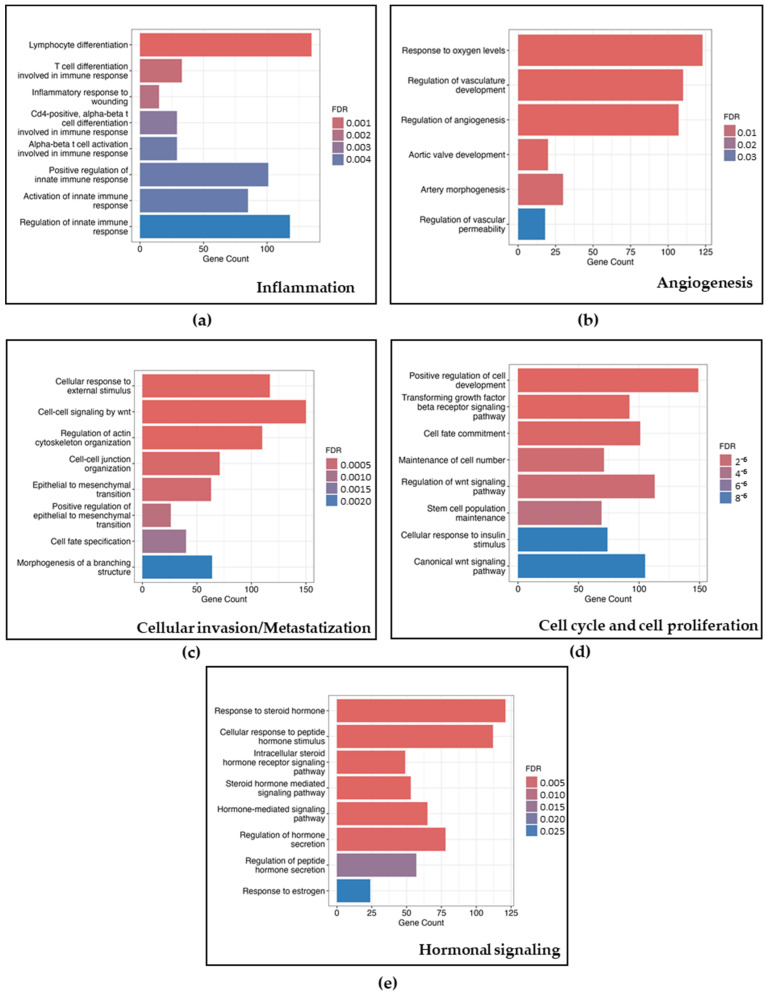
Barplots represent the number of selected target genes of differentially expressed miRNAs, derived from Gene Ontology Biological Processes (GO-BP), in the comparison between endometriosis patients and controls. Pathways were grouped based on their involvement in the main biological processes associated with endometriosis: inflammation (**a**), angiogenesis (**b**), cellular invasion/metastatization (**c**), cell cycle and cell proliferation (**d**), and hormonal signaling (**e**). False Discovery Rate (FDR) is also shown in each barplot.

To explore the pathways that could be influenced by changes in gene expression related to our differentially expressed miRNA target genes between END patients and controls, we conducted a pathway analysis using multiple gene-enrichment algorithms. The complete list of target genes and pathways is shown in Supplementary Tables S1–S5. The identified targets were significantly enriched in various biological processes implicated in END [1,14], such as inflammation, angiogenesis, cellular invasion, cell-cycle/cell proliferation, and estrogen and progesterone hormonal signaling (Supplementary Table S6). Figure 1 and Figure 2 depict gene-concept networks and barplots of genes belonging to different pathways, respectively, for selected GO-BP terms linked to differentially expressed miRNAs. GO-BP images are representative of the results obtained from the other gene-enrichment algorithms.

## 4. Discussion

Although several studies have discovered potential diagnostic biomarkers for END that are easily detectable with non-invasive methods [1,14] none of them have been implemented into clinical practice. Recently, a private company proposed an NGS-based test, called Ziwig Endotest, for the detection of salivary miRNAs for END diagnosis. This test appears to be characterized by good sensitivity and specificity [35], confirming that circulating miRNAs can represent promising diagnostic biomarkers. In the present study, we analyzed serum samples from patients with histologically confirmed END, including OMA and DIE. DIE is characterized by invasive mechanisms, such as high expression of metalloproteinases and activins, increased neuroangiogenesis, altered immune elements, and decreased apoptosis, thus representing a particularly aggressive entity and phenotypically distinct from OMA [36]. Based on this evidence, we evaluated miRNA expression in all END cases compared to controls, as well as DIE and OMA versus controls. First, we investigated a panel of miRNAs that can potentially distinguish all END patients from women without END. Our results revealed five significantly upregulated miRNAs (miR-1249, miR-145-5p, miR-486-5p, miR-485-3p, and miR-26a-5p), and one downregulated miRNA (miR-23a-3p) in END when compared to controls. These had all previously been reported in the literature as related to END, and thus agree with our findings [10,37,38,39,40,41,42].

A study by Neuhausser et al. [37] demonstrated that serum miR-1249-3p was significantly upregulated in END patients compared to the control group. Cosar et al. [38] performed miRNA microarray profiling on serum samples of women with and without END, confirming with RT-qPCR that miR-145-5p was upregulated in END patients. Yang et al. [39] also found miR-145-5p significantly overexpressed in endometriotic tissues compared to healthy endometrium. Similarly, miR-486-5p was upregulated in the peritoneal fluid of END patients, correlating with disease progression [40,41]. Hawkins et al. [42] reported that miR-485-3p was overexpressed in OMA compared to normal endometrium. Both miR-26a-5p and miR-23a-3p were identified in a panel of discriminative miRNAs for distinguishing healthy subjects from END subjects [10].

Additionally, we found other miRNAs (miR-30b-5p, miR-20b-5p, miR-374a-3p, and miR-92b-3p) among the most significantly overexpressed in END compared to controls, which have been associated with inflammation and angiogenesis in various diseases [43,44,45,46,47,48]. These processes are known to be involved in END [1].

From the separate analysis of the two types of END compared to controls, we identified upregulated miRNAs in OMA (miR-142-5p, miR-363-3p and miR-106b-3p) and downregulated miRNAs in DIE (miR-30d-5p, let-7i-5p, miR-340-5p, and miR-29a-3p), which had been previously associated with END in the literature, even if separate comparisons were not reported. Yang et al. [49] described the upregulation of miR-142-5p in eutopic endometrium during the proliferative phase in END patients with infertility. Regarding miR-363-3p expression, Neuhausser et al. [37] showed that this miRNA was also upregulated in serum samples of END patients compared to those of controls. Specifically, miR-106-3p, such as miR-486-5p previously discussed, has been reported to be increased in the peritoneal fluid of END women [41]. 

Regarding downregulated miRNAs in the DIE group, Khalaj et al. [50] found miR-30d-5p significantly reduced in extracellular vesicles from plasma samples of END patients and in ectopic endometriotic lesions compared to healthy controls. Let-7i-5p was significantly downregulated in END plasma samples [51], with a ROC curve showing an AUC of 0.9. Papari et al. [52] using next-generation sequencing and RT-qPCR, found miR-340-5p downregulated in plasma of END patients, a finding confirmed by Bahramy et al. [53]. Finally, miR-29a-3p has also been linked to END [10]. Furthermore, we observed miR-20a-5p to be upregulated and miR-186-5p to be downregulated in DIE compared to controls. Although these miRNAs have not been previously linked to END, they have been reported to play roles in angiogenesis, proliferation, and invasion in cancer [54,55].

To the best of our knowledge, we are the first to have compared circulating miRNA profiles between OMA and DIE. In this comparison, along with previously mentioned miRNAs (miR-23a-3p, let-7i-5p, and miR-30d-5p), we identified miR-103a-3p and miR-17-3p, both previously associated with END [10,52]. MiR-103a-3p was significantly downregulated in END compared to the controls [52], which is consistent with our findings; we observed that this molecule was downregulated in DIE, the most invasive form of END, compared with OMA [10].

In our study, we also conducted a gene enrichment analysis to identify pathways related to the target genes of differentially expressed miRNAs. The results revealed that several target genes are involved in biological pathways related to the pathophysiology of END, including inflammation, angiogenesis, cellular invasion, proliferation, and estrogen/progesterone signaling [56]. These biological processes have been highlighted in previous studies [57,58,59] as key pathways in END. There are several theories regarding the origin of END, and currently, the most common is “retrograde menstruation”. This theory suggests that the onset of endometriotic foci is caused by the migration of menstrual blood into the peritoneal cavity [8]. This leads to the adhesion of endometrial cells to the peritoneum through integrins binding to extracellular matrix adhesion molecules. Menstrual blood contains stem cells capable of differentiating into endometrial cells and may contribute to disease progression, progesterone resistance, and immune system alteration [60]. Consistently, our findings show that target genes are significantly enriched in pathways involving adhesion such as cadherins, integrins, cellular junction, and the regulation of differentiation, maintenance, and pluripotency.

After adhesion, endometriotic cells can infiltrate the peritoneum, leading to tissue reorganization. Moreover, increased estrogen receptor beta (ER-β) promotes cell survival by inhibiting TNF-α mediated apoptosis. Growth factors like EGF, PDGF, and IGF contribute to cell proliferation and lesion progression, while angiogenesis sustains the maintenance of lesions [60] and correlates with neurogenesis, potentially causing chronic pain [8,60]. NF-kB, along with STAT3, p53, Notch, and WNT/β-catenin promote angiogenesis, cell invasion, and immunosuppression in cancer [56]. Consistently, our analysis highlights the importance of cell development, cell invasion, growth factors signaling, and angiogenesis, because target genes were enriched in FGFR, EGF, TNF, IGF1, PDGF, VEGF, STAT3, p53, Notch, WNT, and JACK-STAT pathways in END patients. Moreover, our results support the contribution of the immune system and inflammation in the END microenvironment, as several related pathways, including interleukin (IL-4, IL-6, IL-10, IL-17, IL-37) and chemokine signaling, regulation of innate immune response, lymphocyte and macrophage activation, were found to be significant across the different gene-enrichment algorithms, in agreement with the literature [8,56]. In END patients, hormonal dysregulation, including estrogen (ER) and progesterone receptor (PR) pathways, also emerged as critical factors [56]. Ectopic endometrium exhibits increased ER-β activity, promoting cell survival, inflammation, and epithelial-mesenchymal transition [4], while PR downregulation leads to progesterone resistance, facilitating lesion growth [56]. Our results confirm the relevance of these pathways, including EMT regulation and estradiol/progesterone signaling.

The strengths of our study lie in the fact that, compared to previous investigations aimed at identifying potential diagnostic circulating miRNAs in END that are based on small patient cohorts, ours is a fairly large cohort, which is well-balanced among END patients and controls. Although a larger patient cohort would have been preferable, to the best of our knowledge, our cohort represents one of the largest END patient populations using a high-throughput method [61], with the exception of that from Bendifallah et al., which included 200 patients [19]. Moreover, although it is a retrospective study, the biological samples were collected as part of a multicenter biomarker research effort and were handled and stored in a standardized manner at both centers to minimize pre-analytical bias. Additionally, both the END and the control patients underwent surgery and were confirmed through histological examination, which is critical, as some studies classify healthy patients only based on symptoms, potentially leading to misclassification errors. Another important point in our protocol was the quantification of miRNAs extracted from serum, to ensure that the same amount of RNA was used as input to generate cDNA, thus optimizing the precision of our results. Additionally, we observed significant variability in the amount of small RNA extracted from the same serum volume across different samples. For miRNAs quantification, we used a Qubit kit assay that has been demonstrated in the literature to provide the most accurate measurement of circulating miRNAs [62].

Despite these positive aspects, our research presents some limitations. Firstly, being a retrospective study, data regarding the END stage and the menstrual cycle phases at the time of serum sample collection were not available for most patients, so this information was not included in the analysis of differentially expressed miRNAs. Similarly, data related to adenomyosis was not available for all patients, because hysterectomy was not performed in patients who had surgery for OMA. The high costs associated with the high-throughput method can pose a limitation for diagnostic use; however, the identification of a selected panel of miRNAs in RT-qPCR would allow for a significant reduction in costs.

## 5. Conclusions

In conclusion, our study identified a panel of miRNAs differentially expressed between END and controls, and between DIE and OMA. Our study has also highlighted different pathways related to END biology. These results support the potential involvement of serum miRNAs in the pathophysiology of END and deserve further investigations regarding these molecules as potential non-invasive early diagnostic biomarkers.

## Figures and Tables

**Table 1:** Patients’ characteristics.

	Controls	Endometriosis
	Mean (SD)	Mean (SD)
Age–in years	36.3 (9.0)	36.2 (7.7)
BMI	23.6 (5.0)	21.9 (3.5)
	**N. (%)**	**N. (%)**
Smoking	16 (30.2)	21 (38.9)
Dysmenorrhea	60 (100)	67 (100)
DIE		27 (40.3)
OMA		40 (59.7)
Cystadenoma	25 (41.7)	
Teratoma	13 (21.7)	
Dermoid cysts	4 (6.6)	
Leiomyoma	4 (6.6)	
Other gynecological disorders	4 (6.6)	
No abnormality	10 (16.7)	

SD: Standard Deviation; BMI: Body Mass Index; DIE: Deep infiltrating endometriosis; OMA: endometrioma.

**Table 2:** Differentially expressed miRNAs between endometriosis groups and controls.

miRNA Name	FC (END/CNT)	*p*-Value	FC (OMA/CNT)	*p*-Value	FC (DIE/CNT)	*p*-Value
miR-30b-5p	16.440	**0.0038**	39.742	**<0.001**	-	-
miR-26a-5p	10.002	**0.0048**	14.435	**0.0042**	5.764	0.0984
miR-335-5p	7.194	**0.0482**	8.944	0.0551	-	-
miR-20b-5p	6.691	**0.0035**	5.501	**0.0223**	8.921	**0.0096**
miR-1249-3p	6.162	**0.0062**	5.096	**0.0320**	8.127	**0.0145**
miR-485-3p	3.664	**0.0202**	3.305	0.0616	4.258	**0.0446**
miR-338-3p	3.091	**0.0034**	3.646	**0.0034**	2.418	0.0777
miR-548a-3p	3.010	**0.0431**	-	-	3.372	0.0846
miR-625-3p	2.563	**0.0372**	2.888	**0.0403**	-	-
miR-374a-3p	2.372	**0.0292**	-	-	-	-
miR-145-5p	2.370	**0.0219**	2.256	0.0596	2.548	0.0564
miR-486-5p	1.208	**0.0331**	-	-	1.354	**0.0081**
miR-652-3p	0.798	**0.0152**	-	-	0.709	**0.0043**
miR-23a-3p	0.511	**0.0368**	-	-	0.242	**<0.001**
miR-374a-5p	0.393	**0.0175**	2.552	**0.0385**	0.118	**<0.001**
miR-92b-3p	0.046	**<0.001**	0.176	0.0859	0.006	**<0.001**
miR-133a-3p	-	-	4.724	**0.0239**	-	-
miR-142-5p	-	-	10.464	**0.0298**	-	-
miR-374b-5p	-	-	0.655	**0.0314**	-	-
miR-598-3p	-	-	3.230	**0.0328**	-	-
miR-363-3p	-	-	3.918	**0.0364**	-	-
miR-409-3p	-	-	3.909	**0.0416**	-	-
miR-339-5p	-	-	2.797	**0.0433**	-	-
miR-328-3p	-	-	5.524	**0.0452**	-	-
miR-106b-3p	-	-	4.042	**0.0453**	-	-
miR-361-3p	-	-	2.971	**0.0456**	-	-
miR-30c-5p	-	-	4.273	**0.0490**	-	-
miR-125a-5p	-	-	1.775	0.0507	-	-
miR-30d-5p	-	-	-	-	0.091	**0.0038**
miR-29a-3p	-	-	-	-	0.197	**0.0178**
miR-186-5p	-	-	-	-	0.738	**0.0187**
hsa-let-7i-5p	-	-	-	-	0.045	**0.0241**
miR-421	-	-	-	-	5.846	**0.0397**
miR-20a-5p	-	-	-	-	1.224	**0.0400**
miR-340-5p	-	-	-	-	0.198	**0.0494**

FC (Fold Change) = log_2_RQ_END_/log_2_RQ_CNT_; END (all endometriosis); CNT (controls); DIE (Deep Infiltrating Endometriosis); OMA: endometrioma. Only FC values with *p* < 0.1 are reported. Significant *p*-values are indicated in bold.

**Table 3:** Differentially expressed miRNAs between Deep Infiltrating Endometriosis (DIE) and endometriomas (OMA).

miRNA Name	FC (DIE/OMA)	*p*-Value
miR-20a-5p	1.260	**0.0289**
miR-93-5p	0.818	0.0774
miR-191-5p	0.739	**0.0068**
miR-186-5p	0.730	**0.0235**
miR-22-3p	0.547	0.0775
miR-598-3p	0.331	0.0995
miR-29a-3p	0.297	0.0991
miR-23a-3p	0.287	**0.0043**
miR-103a-3p	0.158	**0.0273**
miR-126-3p	0.143	0.0803
miR-30d-5p	0.134	**0.0235**
miR-374a-5p	0.133	**<0.001**
miR-30b-5p	0.104	0.0870
miR-17-3p	0.099	**0.0374**
miR-92b-3p	0.034	**0.0075**
let-7i-5p	0.032	**0.0192**

FC (Fold Change) = log_2_RQ_DIE_/log_2_RQ_OMA_; DIE (Deep Infiltrating Endometriosis); OMA: endometrioma. Only FC values with *p* < 0.1 are reported. Significant *p*-values are indicated in bold.

## Data Availability

Row data of miRNA expression have been deposited in NCBI’s Gene Expression Omnibus [63] and are accessible through GEO Series accession numbers GSE279435.

## Supplementary Materials

The following supporting information can be downloaded at: https://www.mdpi.com/article/10.3390/biomedicines12102393/s1, Table S1: Target genes of differentially expressed miRNAs between END and controls, and their associated pathway identified through Gene Ontology Biological Processes (GO-BP) annotation enrichment analysis; Table S2: Target genes of differentially expressed miRNAs between END and controls, and their associated pathways identified through Gene Ontology Molecular Function (GO-MF) annotation enrichment analysis; Table S3: Target genes of differentially expressed miRNAs between END and controls, and their associated pathways identified through Kyoto Encyclopedia of Genes and Genomes (KEGG) enrichment analysis; Table S4: Target genes of differentially expressed miRNAs between END and controls and their associated pathways identified through Reactome enrichment analysis; Table S5: Target genes of differentially expressed miRNAs between END and controls and their associated pathways identified through WikiPathways enrichment analysis; Table S6: significantly enriched pathways in END patients grouped according to their function.
